# Nanostructured Graphene Surfaces Promote Different Stages of Bone Cell Differentiation

**DOI:** 10.1007/s40820-018-0198-0

**Published:** 2018-04-17

**Authors:** F. F. Borghi, P. A. Bean, M. D. M. Evans, T. van der Laan, S. Kumar, K. Ostrikov

**Affiliations:** 1 Plasma Nanoscience, School of Physics, The University of Sydney, Sydney, NSW 2006 Australia; 2 CSIRO Manufacturing, P.O. Box 52, North Ryde, NSW 2113 Australia; 3 Brazilian Centre for Physics Research (CBPF), Rua Dr. Xavier Sigaud - 150, Urca, Rio de Janeiro, RJ CEP 22290180 Brazil; 4 School of Chemistry, Physics and Mechanical Engineering, Queensland University of Technology, Brisbane, QLD 4000 Australia; 5 CSIRO-QUT Joint Sustainable Processes and Devices Laboratory, Commonwealth Scientific and Industrial Research Organization, P.O. Box 218, Lindfield, NSW 2070 Australia

**Keywords:** Carbon nanowalls, Graphene films, Bone cell, Cell differentiation, Plasma nanoscience

## Abstract

Nanostructured graphene films were used as platforms for the differentiation of Saos-2 cells into bone-like cells. The films were grown using the plasma-enhanced chemical vapor deposition method, which allowed the production of both vertically and horizontally aligned carbon nanowalls (CNWs). Modifications of the technique allowed control of the density of the CNWs and their orientation after the transfer process. The influence of two different topographies on cell attachment, proliferation, and differentiation was investigated. First, the transferred graphene surfaces were shown to be noncytotoxic and were able to support cell adhesion and growth for over 7 days. Second, early cell differentiation (identified by cellular alkaline phosphatase release) was found to be enhanced on the horizontally aligned CNW surfaces, whereas mineralization (identified by cellular calcium production), a later stage of bone cell differentiation, was stimulated by the presence of the vertical CNWs on the surfaces. These results show that the graphene coatings, grown using the presented method, are biocompatible. And their topographies have an impact on cell behavior, which can be useful in tissue engineering applications.

**Electronic supplementary material:**

The online version of this article (10.1007/s40820-018-0198-0) contains supplementary material, which is available to authorized users.

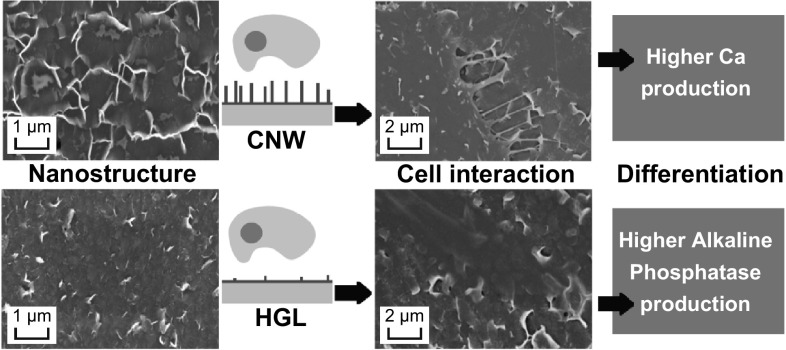

## Highlights

- Horizontally and vertically oriented carbon nanowalls (CNWs) have diverse influences on cell behavior.
- The graphene-based topographies are nontoxic and support cell adhesion and growth.
- Early cell differentiation was observed on the hybrid graphene layer surfaces. Later cell differentiation was stimulated by the CNW films.
- The synergy between the CNWs and differentiation medium provides enhanced mineralization.

## Introduction

The use of nanomaterials for biological control has been a recurring topic of study over the past decades [1–7]. In addition to conventional chemical stimulus strategies, nanomaterials possess reduced-scale features that provide physical control over bacteria and cells [3–8]. Designed nanofeatures are capable of directing and applying forces to cells in order to trigger the activation and/or suppression of genes involved in a number of cellular processes, such as cell adhesion, proliferation, and differentiation (a process where cells become another cell type) [1, 9–12]. Therefore, the use of nanomaterials presents an interesting opportunity to control the fate of cells for the development of biomedical technologies, such as cell therapies and tissue engineering.

Graphene has attracted considerable attention in the biomedical field owing to its unique and specific properties [12–16]. The chemistry of carbon is well known, which makes graphene a useful material for molecular attachment and functionalization. The potential toxicity of graphene and its derivatives (e.g., a solution of graphene nanoplatelets) has been investigated to determine their capabilities for applications in biomedicine [17–20]. For each nanostructure and variations on functionalization and concentration, different results were obtained. Studies on the interactions of graphene coatings/films with cells, using cell-based assays conducted in vitro, have also been reported. Glass and silicon coated with graphene films showed noncytotoxic responses and have been demonstrated to allow the proliferation of diverse types of cells [12, 21–26].

Recent reports have described the use of graphene-based nanostructures to control the differentiation of neural cells [17, 27]. Glass coated with single-layer graphene, in combination with growth factors and proteins, was used to enhance the differentiation of human neural stem cells into neurons [28]. Another study showed that reduced graphene oxide films could support the proliferation of neural stem cells and enhance their specific differentiation [29]. The effects of graphene films on cellular responses were also investigated using human osteoblasts (Saos-2 bone-like cells) and mesenchymal stem cells (MSCs) for bone regeneration applications [19]. Results showed that graphene films presented no cytotoxic effects on these cell types and were supportive of both cell adhesion and proliferation [24]. Interestingly, although the use of graphene films did not influence the overall proliferation rate of MSCs, it did promote their differentiation into the bone pathway more efficiently than did differentiation factors alone [25]. The origin of the enhanced bone differentiation of cells on graphene films was also explored [21–23]. It was found that graphene adsorbed osteogenic inducers that promote the differentiation of the cells into bone-forming cells while suppressing the differentiation of cells toward the adipogenic lineage (fatty tissue). These studies provide evidence of the impact of graphene on bone tissue engineering and its applications.

In addition to the chemical interaction between graphene and biological molecules, some of these reports mentioned the presence of topographical features on the graphene surface, such as wrinkles and ripples, as being a contributing factor in the cellular interactions [18, 21, 23, 25, 29, 30]. Despite the potential impact of these topographical nanofeatures in carbon-based materials, their intentional use to control cellular behavior has been poorly explored. The few examples include the use of vertically aligned carbon nanotubes and carbon nanowalls (CNWs) [31, 32]. However, to the authors’ best knowledge, no published study has been specifically designed to compare the effects of graphene-based topographies containing nanoscaled features (e.g., CNWs) with those of horizontally oriented graphene on the control of the cellular response.

Among graphene-based materials, CNWs possess a unique morphology, being composed of few-layer graphene sheets oriented vertically on a substrate, which gives them an inherently high aspect ratio [33–35]. The nanowalls have the advantages of carbon’s ability to bind biologically important molecules, specific properties of graphene-like materials, and the ability to provide topographical cues at the cellular level. The vertical nature and inherent topological effects are of particular importance as platforms for cellular attachment and for control of cells via mechanical stress. CNWs can be produced by a simple, reproducible, technologically sustainable, and unique plasma process [36–38]. This plasma-enhanced chemical vapor deposition (PECVD) technique has demonstrated improved control over the CNW structures at relatively low temperatures, and it is also possible to fabricate films with horizontal and vertical CNWs of a similar thickness and size and therefore similar (nontopological) properties [32, 36, 39]. Furthermore, this PECVD process allows chemical-free transfer of the CNW films onto the desired substrates, thereby reducing the risk of contamination with hazardous chemicals, which is of great importance when considering biological interactions [38].

In this study, we investigated the ability of different graphene-like films produced by the PECVD system to control the differentiation of Saos-2 cells into bone-like cells in vitro. The CNW films were compared with horizontally oriented graphene sheets that were produced using the same PECVD method with the same composition. This ensured similarity of the chemical properties on both surfaces, with the nanotopography being the only difference between them. The impact of defined graphene-surface nanotopography on cellular processes (including cell adhesion, proliferation, and differentiation stages) was examined using quantitative cell-based assays with the Saos-2 cell line, which is a human bone cell line that is known to differentiate in vitro when provided with specific culture medium supplements.

## Experimental

### Growth and Chemical-Free Transfer of the Graphene Nanostructures

The growth of the graphene nanostructures was carried out in a radio-frequency (RF) inductively coupled plasma CVD system, as described in previous reports [35–38, 40]. A piece of copper foil (99.95%, Alfa Aesar) of 4 × 4 cm^2^ dimensions was used as the growth substrate and placed on a stage in the plasma chamber. First, a gas mixture of 10 sccm argon (Ar) and 20 sccm hydrogen (H_2_) was fed into the chamber, and a pressure of 1.5 Pa was set. The plasma was then produced using 750 W from an RF power supply and a matching network. The copper foil was treated for 3 min with the H_2_–Ar mixture, following which methane was fed into the chamber for 6 min. The density of the graphene nanowalls was controlled by varying the H_2_ flow rate supplied during deposition. For this work, a high H_2_ content (80 sccm) was used to produce fewer and thinner CNWs, whereas a lower H_2_ content (20 sccm) generated denser and thicker CNWs on the copper foil. No external substrate heating was used; however, the substrate temperature reaches ~ 250 °C owing to plasma heating effects [38]. Next, all produced CNW films were decoupled from the copper foil using deionized water. The foil was placed in the water and sink while the graphene film was left floating on the water surface. Subsequently, the films were transferred onto Thermanox™ coverslips for biological characterization. These substrates (made by Nunc and purchased from Thermo Fisher Scientific, Australia) are made from a hydrophilic polyester film with very low oxygen content and are highly resistant to solvents. Uncoated Thermanox™ coverslips were used as the experimental control. Therefore, by simply controlling the H_2_ content, the same production and transfer processes yielded two different topographies. The first comprised a dense CNW surface, and the second consisted of thin CNWs oriented horizontally with very few standing CNW sheets [hereafter named as hybrid graphene layers (HGLs)].

### Material Characterization

The topography of the graphene nanostructures was studied by field-emission scanning electron microscopy (FE-SEM), and images were obtained with a Zeiss Auriga microscope operated with 5 keV electron beam energy and an InLens secondary electron detector. Raman spectroscopy was carried out on the graphene nanostructures, using a Renishaw inVia spectrometer with laser excitation at 514 nm (Ar laser) and a probing spot size of ~ 1 µm^2^. The hydrophobicity of the samples and of the uncoated coverslip control was determined using contact angle measurement at room temperature, where a high-resolution optical camera and free image analysis software (ImageJ) were used to obtain the photographs and angles between a water droplet and the samples.

### Sample Sterilization

All graphene films on Thermanox™ coverslips produced for the cell-based assays were sterilized in 70% ethanol overnight and then rinsed with phosphate-buffered saline (PBS) the following day. All cell culture procedures after this substrate sterilization procedure were conducted in a biohazard hood, using aseptic techniques.

### Cell Adhesion and Proliferation

Cell adhesion and cell proliferation assays were conducted on the graphene films as well as on uncoated Thermanox™ coverslips and tissue culture polystyrene (TCPS) as controls. The cell adhesion studies were run over 24 and 48 h to examine the early adhesion and spreading behavior of cells, which were examined by light microscopy using phalloidin staining and also by SEM. The cell proliferation assay was conducted over 7 days to track the growth kinetics of the cells. All cell-based assays were conducted using a human bone-derived cell line (Saos-2), which is recognized to have the capacity to differentiate into mature bone-like cells that produce alkaline phosphatase (ALP) and a bone-like mineralized matrix when cultured under the appropriate mineralizing/differentiating (osteogenic) conditions [41, 42]. The Saos-2 cells were routinely grown in Dulbecco’s modified Eagle’s medium (DMEM)/nutrient mixture F-12 supplemented with 100 U mL^−1^ penicillin, 100 µg mL^−1^ streptomycin, 20 mM l-glutamine (all from Gibco, Australia), and 10% fetal bovine serum (FBS; Bovagen, Australia). For all cell adhesion and cell proliferation assays, triplicate samples of each graphene sample and control surfaces were placed in individual wells of a 24-well TCPS plate (Corning, Australia). Saos-2 cells were then seeded onto each surface at a concentration of 3 × 10^4^ cells mL^−1^ (1 mL of cell suspension per well). The plates were maintained in an incubator at 37 °C in a humidified atmosphere containing 5% CO_2_ until the predetermined time points for each assay.

#### Cell Adhesion Study

The cell adhesion study was designed to monitor the early adhesion and spreading behavior of cells on the test surfaces to 24 and 48 h time points, and samples were evaluated using either phalloidin staining to identify the actin filaments of the cell cytoskeleton or SEM to probe the cell–material interactions at higher magnifications. Samples for phalloidin staining were transferred to the wells of a fresh TCPS plate and fixed in 4% formol saline for 30 min at room temperature. The cells were then permeabilized in 0.1% Triton X-100/PBS for 5 min at room temperature, blocked in 1% BSA/PBS for 60 min, and then stained with a 1/40 dilution of Alexa Fluor 647 phalloidin (Thermo Fisher Scientific, Australia) for 30 min at room temperature in the dark. Finally, the substrates were washed twice with PBS and the adherent cells were visualized under a fluorescence microscope with a TRITC filter (Nikon IIi) and images were captured with a digital camera. Samples for SEM were washed in PBS and fixed with 10% paraformaldehyde for 30 min at room temperature and then washed in PBS before dehydration. The samples were dehydrated using a series of ethanol solutions (increasing from 30 to 100% and repeating the last step) for 10 min at each step. Then, the samples were soaked in hexamethyldisilazane (Sigma, USA) for 2 min at room temperature and allowed to dry overnight in a desiccator. Finally, the samples were mounted on SEM stubs, coated with 10 nm gold film, and placed in a desiccator until viewed by SEM (same as for sample characterization).

#### Cell Proliferation Assay

The cell proliferation assay was designed to measure the growth of cells over a 7-day period at the predetermined time points of days 1, 3, and 7. One extra sample of each type of the graphene and control surfaces was included for cell staining and imaging on day 7 to check the morphology of cells on each substrate. At each of the three time points, one plate was removed and samples were transferred to the wells of a fresh TCPS plate for MTT assay to measure the viability/number of adherent cells on each graphene sample. An MTT assay is a colorimetric assay used to measure cell viability, in which the yellow-colored 3-(4, 5-dimethylthiazol-2-yl)-2, 5-diphenyltetrazolium bromide (MTT) is converted into dark-blue water-insoluble formazan crystals by the activity of mitochondrial dehydrogenases in living cells. After transferring samples to fresh plates, 10 µL of Thiazolyl Blue Tetrazolium Bromide reagent (MTT; Sigma #M5655) at a final concentration of 0.5 mg mL^−1^ in fresh serum-free medium was added to each well of the plates, which were then returned to the incubator for 4 h. After the incubation in MTT, the medium in each well was replaced with 1 mL of dimethyl sulfoxide, and the plates were shaken for 10 min to dissolve the formazan product. A 100-µL sample was then taken from each well and transferred to the wells of a 96-well plate for spectrophotometric determination at 595 nm using a plate reader (SpectraMax, Molecular Devices, USA). At day 7, the extra samples included for cell morphology examination were rinsed in PBS, and the adherent cells were fixed in 4% formol saline for 30 min at room temperature. After rinsing in PBS and staining with 0.4% trypan blue for 15 min at room temperature, the cells were rinsed in PBS and inverted onto a glass microscope slide in glycerol:PBS (1:1). Samples were viewed using both phase contrast and fluorescence microscopes set on FITC wavelength, as the Thermanox™ material is autofluorescent at FITC wavelength, allowing darkly stained cells adhered to the opaque substrates to be visualized and imaged.

### Differentiation/Mineralization Assays

This experiment was designed to test the ability of the graphene films to support the differentiation of bone cells, with evidence of mineralization over a longer time period. Cell-based assays using Saos-2 cells were used to assess cell differentiation by measuring the cellular production of the ALP enzyme and calcium over a 25-day time period in culture. For the differentiation assay, each of the Thermanox™ coverslips coated with a graphene film was placed into an individual well of a 12-well TCPS culture plate. Thermanox™ coverslips without graphene films and TCPS wells with no substrates were also included as assay controls. Saos-2 cells were seeded onto the substrates and controls at a concentration of 5 × 10^4^ cells cm^−2^ in standard Saos-2 culture medium, and the plates were then placed in a 37 °C incubator supplied with 5% CO_2_ in humidified air for 72 h to allow cell attachment and growth. After 72 h, the culture medium was removed and replaced with either (1) standard culture medium (DMEM/F12 containing 10% FBS) as an undifferentiated control culture regime, or (2) a differentiation/osteogenic-specific culture medium containing additives specifically known to promote cell maturation/differentiation along the osteogenic/bone lineage (DMEM/F12 containing 10% FBS, 100 U mL^−1^ penicillin, 100 µg mL^−1^ streptomycin, and 20 mM l-glutamine and supplemented with 1.5 mg mL^−1^ β-glycerophosphate, 40 ng mL^−1^ dexamethasone, and 20 µg mL^−1^ ascorbic acid). These two culture regimes were continued in both series of plates for the rest of the 25-day assay period, with the medium replenished every 3–4 days. On day 25, samples maintained on these culture regimes were assayed for the ALP and calcium contents in adherent cells (as described in Sects. 2.5.1, 2.5.2 below).

An extra complete set of samples and controls was established to run in parallel with the differentiation assay so that the growth of the Saos-2 cells on each graphene-coated substrate at day 25 could be evaluated by standard MTT assay (as described in Sect. 2.4.2). This enabled the differentiation/mineralization data (ALP and calcium) to be adjusted according to the number of cells attached to each test substrate and TCPS control at day 25.

#### Alkaline Phosphatase Assay

ALP is a by-product enzyme of active bone formation, which means its level increases when the bone matrix is being formed. Induction of ALP can thus be used to predict bone formation in vitro as well as to measure early-stage cell differentiation [43]. ALP production was measured in cells attached to the graphene surfaces and controls at 25 days, using a quantitative assay based on *p*-nitrophenyl phosphate (*p*-NPP; Sigma #N-1891). In brief, 500 µL of *p*-NPP at a concentration of 1.0 mg mL^−1^ in 0.1 M glycine buffer was added to each well containing a Thermanox™ coverslip, and the plate was shaken at 37 °C for 60 min. Then, the reaction was neutralized by the addition of 500 µL of 1 M NaOH per well. The sample sets were compared against a standard curve prepared from a 10 µmol mL^−1^ stock solution of *p*-NPP diluted in 0.1 M glycine buffer to create eight *p*-NPP standards ranging from dilutions of 1/5–1/640. The optical density of the wells containing test samples and standards was determined spectrophotometrically at 405 nm using the Spectramax plate reader. The results were normalized to take into account the number of cells present (cell density) on each surface, as determined by the MTT assay conducted in parallel with the differentiation assay over 25 days.

#### Calcium Assay

The calcium produced by bone cells as they differentiated/matured was used to identify mineral production by the cells attached to the test substrates after 25 days in culture. A calcium-sensitive dye, Arsenazo III, was used in this quantitative assay. The Thermanox™ coverslips coated with graphene films were individually transferred to the wells of culture plates, and 500 µL of 0.6 M HCl was added to each well. The plates were then shaken overnight at room temperature to permeabilize the cells and release the calcium. On the next day, the reaction was neutralized by the addition of 100 µL of 3 M NaOH per well. A 20-µL aliquot was then taken from each well and mixed with 200 µL of Arsenazo III reagent (Sigma #11090) at a concentration of 0.15 mg mL^−1^. The dye forms a complex with the calcium, resulting in a color change. The optical density of the test sample and standard wells was determined spectrophotometrically at 595 nm using the Spectramax plate reader. The sample sets were compared against a standard curve, which was prepared from a 1 mg mL^−1^ CaCl_2_ stock solution diluted in H_2_O to create eight CaCl_2_ standards ranging from dilutions of 1/100 to 0 µg mL^−1^. The results were normalized to take into account the number of cells present (cell density) on each surface, as determined by the MTT assay conducted in parallel with the differentiation assay over the 25-day period.

### Statistical Analysis

Unless otherwise specified, the biological assays were performed in triplicates. The data are reported as the mean ± standard deviation, where the statistical significance of differences (*p* < 0.05) was determined with Student’s *t* test.

## Results and Discussion

### Surface Morphology of Graphene Surfaces

**Fig. 1:**
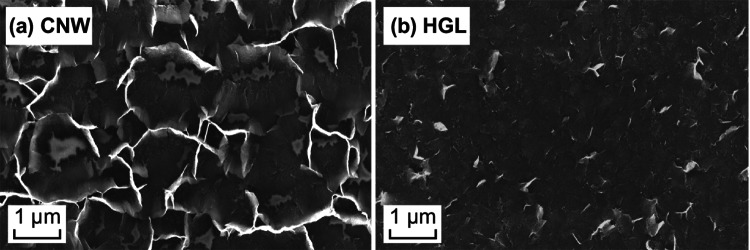
SEM images of the graphene-like samples. **a** CNW, **b** HGL nanostructures on Thermanox™ coverslips. These images show the clearly visible edges of the CNWs and the edges of the horizontally aligned CNWs

The results of FE-SEM analysis of the surface morphology of the CNW and HGL films that were produced and transferred to the coverslips are presented in Fig. 1a, b, respectively. The CNW films consisted of thin graphene-like walls (up to 10 nm thick) that were perpendicular to the substrate in a spaced array of 2-µm intervals on average (Fig. 1a). This type of graphene morphology represents a plasma-unique assembly of three-dimensional graphene, creating a scaffold that offers binding support for cells and an open-surface arrangement for molecular exchange. It possesses graphene structures at a similar order of magnitude to that of the organelles present on the cell surface and is also capable of accommodating smaller cells in between its walls. Furthermore, the process of formation and functionalization of these CNWs has been well studied, and the control of features widens their application capabilities [39, 44].

The HGL surfaces were created using the same process as used for the CNWs, to maintain the chemical composition; however, a lower relative concentration of methane was used. This approach yielded similar nanostructures prior to the transfer, made of thinner vertically aligned walls that collapsed onto the substrate during the transfer process (Figs. S1 and S2), leaving the few-layered graphene sheets (10 nm thick, 1 µm^2^ of area) lying horizontally on the substrate surface (Fig. 1b). However, these horizontal graphene sheets do not collapse in a uniform and completely flat manner, as can be seen in Fig. 1b where some of the sheets have edges raised from the surface that appear brighter in the image (owing to charging effects) and show some of the sheet boundaries. Nanostructures presenting a similar thickness and crystalline structure have been produced by the same process and extensively characterized by van der Laan and by Pineda et al. [36–38, 40, 45–47]. It should be noted that techniques have been developed to produce horizontally oriented CNWs by blinding the electromagnetic field of the plasma process, which follows a different growth mechanism [39].

These horizontally aligned, texturized surfaces were used to compare with the CNW surfaces, as they provided both a reduced graphene surface area (with only one plane available) and fewer topographical features for biological interactions while retaining the interactivity with the complex chemical system of the culture medium. These topographical differences were used to discriminate the influence of the mechanical anchoring of the cells from the purely chemical influence of the presence of graphene.

### Surface Composition of Graphene Surfaces

**Fig. 2:**
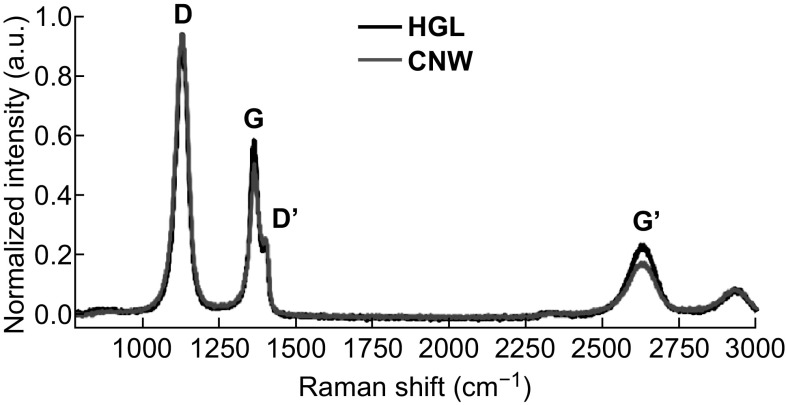
Raman spectroscopy of the CNW (in red) and HGL (in black) samples. Both spectra feature the graphene peaks at about 1350, 1580, and 2690 cm^−1^. The shoulder peaks at ~ 1620 cm^−1^ in the spectra, which are related to thicker graphene sheets, are more evident on the CNW sample (A color version of this figure can be viewed online)

Raman spectroscopy was used to evaluate the similarities between the surface compositions of the CNW and HGL films. The Raman signals (Fig. 2) verified the growth of the graphene-like films through the presence of the graphene-specific bands of disorder (D at 1350 cm^−1^), graphite (G at 1580 cm^−1^), and second-order disorders (G′ at 2690 cm^−1^). The D-band is related to the crystallite size effect and structural defects in the *sp*^2^-carbon. The G-band represents the in-plane vibrational *E*_2g_ mode of the hybridized *sp*^2^-carbon. The defect-related peaks are second-order features attributed to the stacking of graphene sheets and the high edge density [39, 41, 46–49].

The G peak (graphitic peak) was narrow for both graphene samples, whereas the D peak (defect peak), related to the predominance of graphene edges on the surface, was slightly higher for the CNW films owing to the edges of the vertically aligned graphene sheets. These results showed a higher intensity ratio between the D and G peaks (*I*_D_/*I*_G_) on the CNW samples (1.85) than on the HGL samples (*I*_D_/*I*_G_ of 1.61), which was in agreement with the literature on graphene and CNWs [20–22, 39, 49]. However, the use of a more defectuous graphene film is justified in order to be able to compare both nanotopographies grown by the same technique. Importantly, although the concentration of methane used for the deposition influenced the morphology of the graphene films, the samples nevertheless retained a very similar composition.

**Fig. 3:**
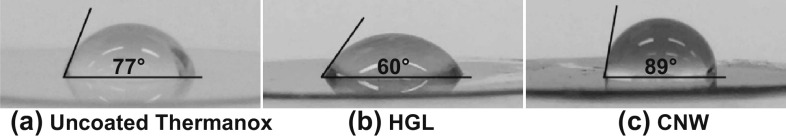
Photographs used for contact angle measurements of **a** uncoated Thermanox™, and Thermanox™ coated with **b** HGL or **c** CNWs. These images show the effect on the surface due to the presence of the nanostructures, turning more hydrophilic as a result of the horizontally aligned CNW structures (60°) and less hydrophilic because of the CNW edges (89°)

The results of the contact angle measurement, conducted using the sessile drop method, are shown in Fig. 3. The uncoated Thermanox™ coverslips presented a wettable surface (contact angle of 77°), which became more hydrophilic when coated with the HGL structures (contact angle of 60°). Although planar graphene is generally hydrophobic, the exposed edges of the horizontally aligned CNWs on the HGL sample may increase its surface energy. When coated with CNW films, the resultant textured surface was less hydrophilic, with a contact angle of 89°, which is a general result found in the literature and may be attributed to air pockets between the CNWs [50]. These results show the effects of the graphene-based nanotopographies on the wettability of the naturally hydrophobic graphene, which may influence the proliferation of cells as a result of the adhesion of important biomolecules [20–22, 51, 52].

### Adhesion and Proliferation of Saos-2 on the Graphene Surfaces

**Fig. 4:**
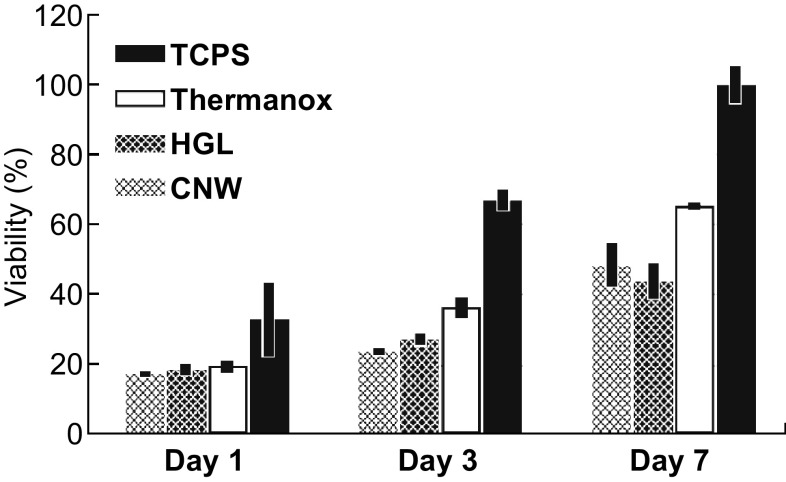
Proliferation assay showing the growth of Saos-2 cells on the CNW, HGL, and control surfaces over a 7-day period, as measured by MTT assay (based on triplicate samples of each surface at each of the three time points, with outcomes normalized against the cell viability level on TCPS at 7 days)

The growth of cells on the CNW and HGL surfaces, as well as on the uncoated Thermanox™ control surface, was measured by MTT assay at three time points over the total assay time of 7 days, with resulting data normalized to the TCPS control surface at day 7 (Fig. 4). There was no significant difference in the number of cells attached to the graphene-coated surfaces and Thermanox™ control surfaces on day 1. However, by day 3, there were fewer cells adhered to either of the graphene-like-coated substrates than there were to the uncoated Thermanox™ control. This trend persisted to day 7, with cell numbers increasing on all surfaces but with a lower rate of cell proliferation on both graphene surfaces. The cell count was approximately 20–25% less on the graphene-like surfaces than on the uncoated Thermanox™ control. This was an expected outcome, considering that the Thermanox™ coverslips are specifically designed to promote cell adhesion and proliferation. Similar lower proliferation outcomes had been observed in other works, especially when polymeric substrates were used [21, 23, 24, 26]. The pertinent outcome here is that both of the graphene-like-coated surfaces supported levels of initial cell adhesion and a steady cell proliferation rate that were at least 75% of the level found on the uncoated Thermanox™ control over the 7-day period of the assay.

**Fig. 5:**
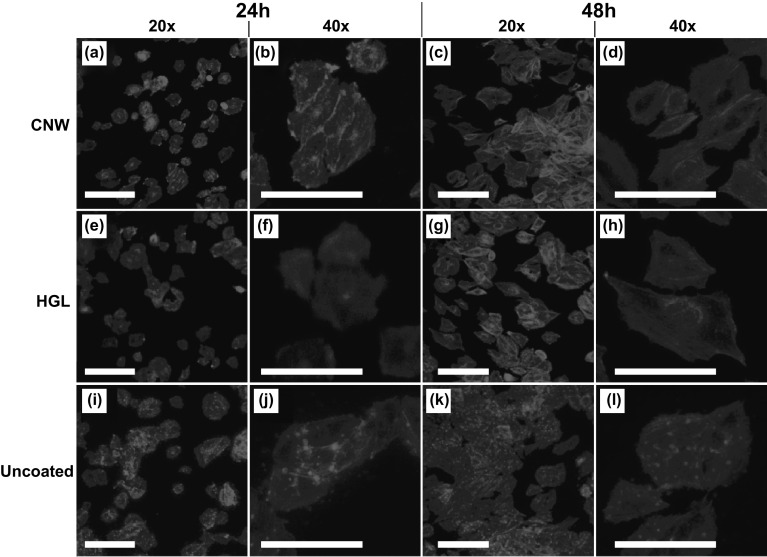
Representative fluorescent images showing the morphology of Saos-2 cells stained with phalloidin and attached to the **a**, **b** CNW surface@24 h (× 20 and × 40), **c**, **d** CNW surface@48 h (× 20 and × 40), **e**, **f** HGL@24 h (× 20 and × 40), **g**, **h** HGL@48 h (× 20 and × 40), **i**, **j** uncoated Thermanox@24 h (× 20 and × 40), and **k**, **l** uncoated Thermanox@48 h (× 20 and × 40). All scale bars are 10 µm (A color version of this figure can be viewed online)

The proliferation results were confirmed by the fluorescence microscopy images of the cells over the surfaces (Fig. 5), which reflected the cellular response to the surface topography properties of the CNWs and HGL compared with that to the smooth control surface (Thermanox™). The adhesion of Saos-2 cells was studied at 24 and 48 h during the proliferation assay by staining adherent cells on the samples with phalloidin. This assay provides some insight into the interactions of the actin cytoskeleton in the spreading behavior of cells on the graphene and control surfaces (Fig. 5). Although the cell attachment levels on the CNWs were similar to the ones found on the HGL samples, there were some differences in the morphology of the adherent cells, presumably due to the cellular response to the different topographical cues available from the two graphene surfaces. The HGL provided the cells with a biocompatible surface and the cells were spread, but the cell shape was not well defined and there were very few evident focal adhesion points between the cells and the surface. On the other hand, cells on the CNW surfaces were well spread with more focal adhesion points and, in this way, were similar to the appearance of cells on the control surface (Thermanox™). This was most noticeable at the 24 h time point, indicating that cells could spread more rapidly on CNW than on HGL surfaces (Fig. 5a, b, e, f, i, j). By 48 h, the majority of cells on the CNW surface had continued to spread, but some appeared to be smaller and may have been confined to the topographical features of this surface.

The proliferation assay, combined with the early adhesion test and the contact angle measurement (Figs. 3–5 and S3), showed a certain degree of similarity of the cellular response on the studied surfaces. This similarity may rely on a combination of the different surface chemistries, topographies, and protein adsorption patterns. Although more wettable surfaces, like CNWs and Thermanox™, are expected to provide better cell attachment, their chemistry and topography may be playing combined roles to provide similar results [51–54]. Graphene-coated surfaces have been reported to allow a high adsorption of serum proteins (due to *π*-electron interaction), which contain extracellular matrix proteins (e.g., albumin, fibronectin, vitronectin); this may explain the cellular behavior on the graphene-coated samples and suggests a similar profile of protein adsorption [21, 23]. Although the difference in the wettability of each surface is also expected to affect cell attachment, their different topographies combined with the protein adsorption may be working synergistically to promote a similar cellular response [51]. This hypothesis of the mechanism involved remains to be tested.

**Fig. 6:**
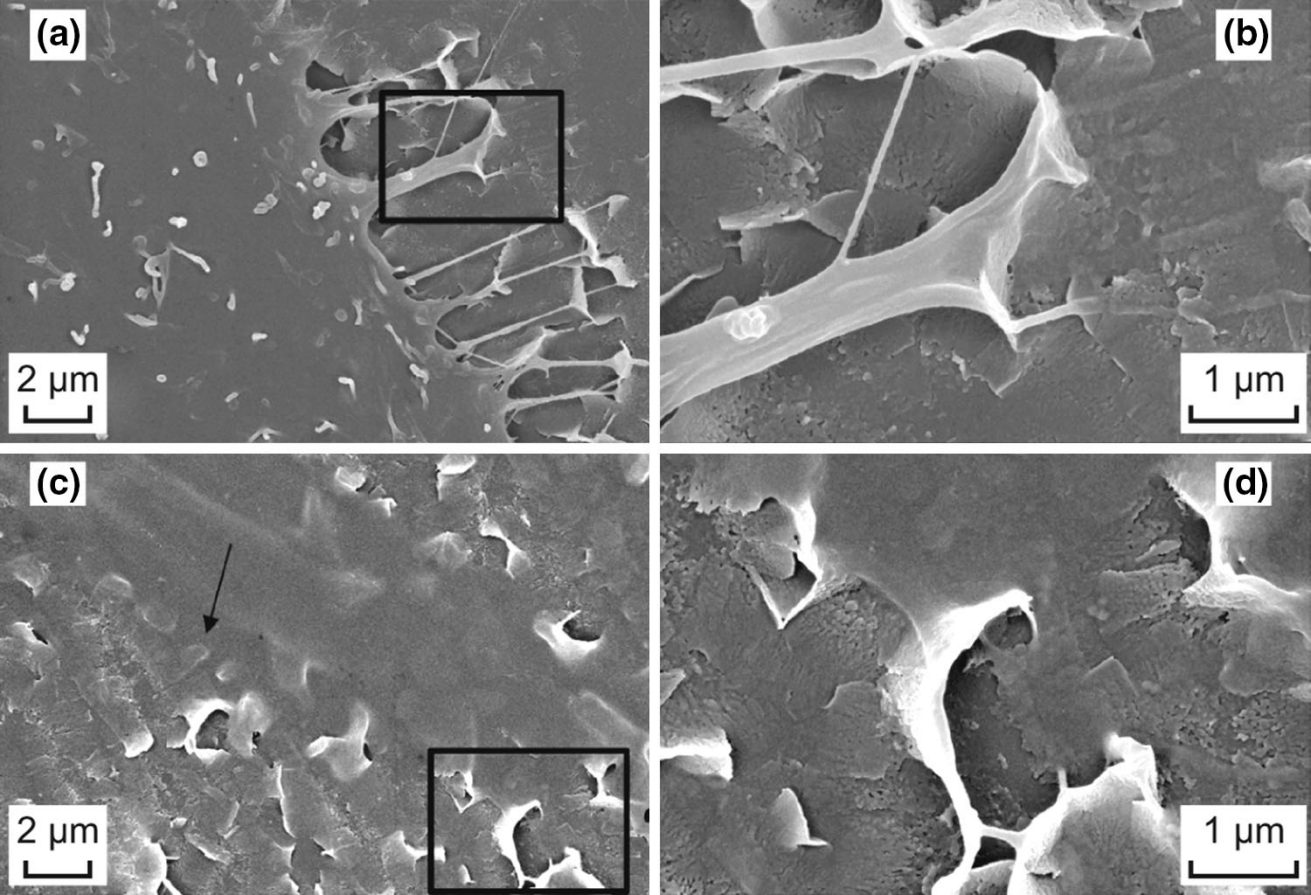
SEM images showing details of the cell interactions with the surfaces after 48 h of incubation. **a** A cell on the CNW surface exhibiting several elongated extensions connected to surrounding nanowalls, presented in detail in **b**. **c** A cell on the HGL surface presenting little interaction with the surface (arrow) but on some nanofeatures, presented in detail in **d**

The SEM images revealed more details about the interaction of the cells with the graphene surfaces during the spreading process (Fig. 6), where it was evident that cells attached to the CNW surface had fine cytoplasmic processes extending across the surface and attaching to the CNWs as they spread. This confirmed that the cells could detect the topographical features that the CNW surface presented to them. As was previously observed with similar CNW structures, adherent cells can be encouraged to migrate and proliferate, or be inhibited to adhere, depending on the cell type and functionalization of the CNW edge [51, 52, 54, 55]. The adhesion mechanism proposes an extension-latch-and-release process, similar to the observations made in a lower density CNWs like the ones produced in this study [55]. In other previous studies, graphene has been shown to present topographical features (wrinkles, ripples, and grids) that influenced protein adsorption, and cell adhesion, proliferation, and differentiation [21–25]. These out-of-plane deformations were pointed to enhance the reactivity of the surface and promote an accelerated differentiation of MSCs by inducing physical stress [22, 23]. A direct comparison between rippled graphene and carbon-based flat surfaces directs the attention to the importance of this deformation in cell differentiation [25]. Therefore, the topographical features of the CNWs are important factors to be considered in influencing the cellular behavior toward specific differentiation paths, because of their physical interaction with cells and increased chemical interaction with relevant proteins.

### Differentiation/Mineralization Assays of Saos-2 on the Graphene Surfaces

**Fig. 7:**
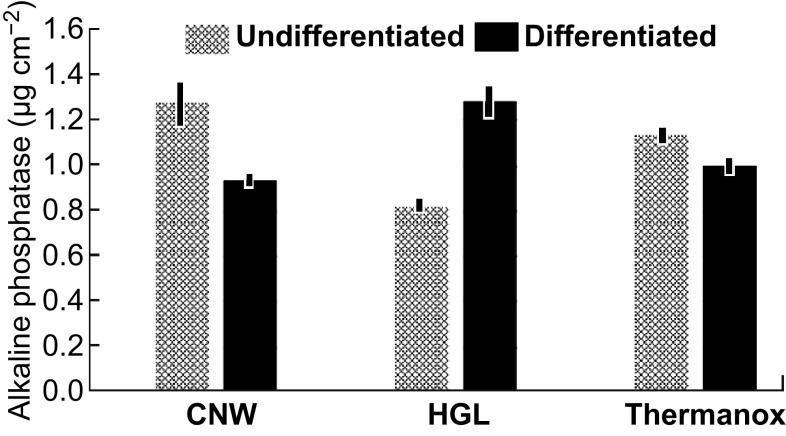
Outcomes of the differentiation/mineralization assays showing the production of ALP by cells attached to the graphene and control surfaces. Data are based on the mean (± standard deviation) of triplicate wells for each surface, corrected for cell density (as measured by MTT assay conducted in parallel with the differentiation assay)

The process of Saos-2 differentiation on the graphene and control substrates was monitored over 25 days by measuring the ALP and calcium produced by the adherent cells. Data from both the ALP and calcium assays were corrected for cell density (as measured by the MTT assay performed in parallel with the differentiation assay over 25 days). The ALP assay, the outcomes of which is an indicator of the early stage of the cellular differentiation process, showed that the graphene surfaces supported the enzyme’s production at a level equivalent to or better than the uncoated Thermanox™ control when cells were maintained under the differentiation/osteogenic culture regime (Fig. 7, dark columns). Under the differentiation/osteogenic conditions, cells attached to the HGL surfaces produced approximately 25% more ALP than did cells on the CNW or uncoated Thermanox™ control surfaces.

This outcome indicated that the cells on the HGL surfaces were in an earlier stage of the differentiation process and were more inclined toward proliferation compared with their counterparts on the CNW surfaces that were more actively differentiating. The higher levels of ALP produced by cells on the HGL surfaces revealed that the cells were in a less-differentiated state. This may be explained by the ability of graphene to adsorb important molecules known to induce cell proliferation, as shown by Lee et al. [21]. In the mentioned study, graphene was shown to preconcentrate dexamethasone and β-glycerophosphate (usual osteogenic growth factors), which was attributed to the *π*–*π* interaction between the aromatic moieties of the molecules and the graphene plane [21]. Similarly, other graphene-based topographical features (grids) presented highly accelerated differentiation that was also attributed to their ability to adsorb chemical inducers [23]. Furthermore, our results of a synergistic association of graphene and growth factors yielded higher differentiation, in accordance with previous reports [23]. Notably, the aforementioned effects for the HGL samples could be further enhanced on the CNW samples owing to their higher surface area and edge reactivity, where more chemical inducers could be adsorbed onto the vertical basal planes or could react to the chemical terminations of the CNW edges.

**Fig. 8:**
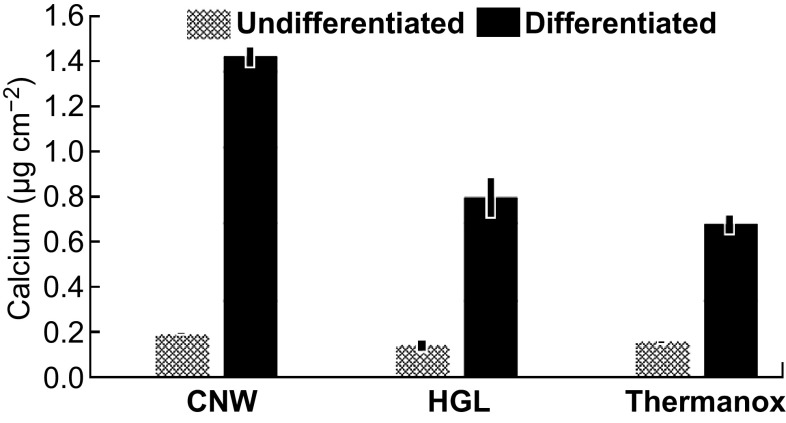
Outcomes of the differentiation/mineralization assay showing the production of calcium by cells attached to the graphene and control surfaces. Data are based on the mean (± standard deviation) of triplicate wells for each surface, corrected for cell density (as measured by MTT assay conducted in parallel with the differentiation assay)

The calcium assay showed that cells attached to the CNW-coated surface produced twice as much calcium as that produced by cells on either the HGL samples or the uncoated Thermanox™ control surface when cultured under differentiation/osteogenic conditions (Fig. 8, black columns). Since calcium production is a measure of the later stage of cellular differentiation, where the mineral associated with bone is forming, this outcome showed that the CNW surfaces significantly promoted the mineralization aspect of the bone cell differentiation process. This observation is consistent with the surface morphology of the as-produced graphene films (Fig. 1). The planar graphene film on the coverslips comprised nano-to-micro features, including wrinkles and ripples, which stimulated cell differentiation and resulted in higher ALP production. Most of these features on the films originated from (1) the difference in the thermal expansion coefficients of the graphene film and the native copper catalyst during their decoupling in water, and (2) trapped water between the film and coverslip during the transfer process.

However, the randomly distributed nano-to-micro-vertical nanowalls on the CNW samples are a distinct topography (Fig. 1a) with noticeable disorder (Fig. 2) that stimulates cell differentiation, resulting in higher calcium production. It should be noted that the stimulated cell differentiation was solely related to the graphene morphology, as our graphene transfer process eliminated any chemical contamination. These results reinforce the ability of graphene-based nanotopographies to control the cell differentiation process by serving as anchoring points for cells.

The calcium release results were consistent with the ALP production by the cells, as the higher production of the enzyme on the HGL samples indicates that the cells had started the differentiation process but did not proceed with mineralization, producing less calcium overall. On the CNW surfaces, the topographical cues enhanced calcium production, showing that the cells were in a later stage of differentiation, and the production of ALP was therefore reduced (as identified in the enzyme assay).

## Conclusion

The data from our adhesion and proliferation studies showed that cultured human bone cells could attach to and grow on both CNW and HGL nanostructures. Furthermore, the adhesion and proliferation outcomes were within approximately 75% of the level that occurred on the control Thermanox™ surface, a material specifically engineered to promote these cellular processes. Cells also spread on both the HGL and CNW surfaces, with some subtle but evident differences between the two surfaces in that aspect of cell behavior. Differentiation data showed that the cells were significantly affected by the topographical cues of the underlying graphene surfaces. The HGL surface maintained cells in a less-advanced differentiation state (as determined by ALP data), whereas the CNWs promoted the later stage of the cell differentiation process, as marked by the cellular mineral production (demonstrated by the calcium data). In conclusion, we have shown that graphene-coated surfaces are biocompatible and that the topography of the graphene coating can have a profound influence on the long-term behavior of cells, in particular the cellular differentiation and mineralization process involved in the generation of new bone (osteogenesis). The optimization of these designed topographical features based on graphene-coated surfaces using PECVD may lead to improved outcomes in tissue engineering.

## Electronic supplementary material

Below is the link to the electronic supplementary material.

Supplementary material 1 (DOCX 2265 kb)
