# Supplementary material for: Nanostructured Graphene Surfaces Promote Different Stages of Bone Cell Differentiation
Source: Nanomicro Lett. 2018 Apr 17;10(3):47. doi: 10.1007/s40820-018-0198-0 (PMC6199093; doi:10.1007/s40820-018-0198-0)
Supplement: Supplementary file 1 — Supplementary material 1 (DOCX 2265 kb) [file 40820_2018_198_MOESM1_ESM.docx]

Supporting Information for

**Nanostructured Graphene Surfaces Promote Different Stages of Bone Cell Differentiation**

F.F. Borghi^1,2,3^, P.A. Bean^2^, M.D.M. Evans^2^, T. van der Laan^4,5^, S. Kumar^4,5^, K. Ostrikov^1,4,5,^*

^1^Plasma Nanoscience, School of Physics, The University of Sydney, Sydney NSW 2006, Australia

^2^CSIRO Manufacturing, PO Box 52 North Ryde NSW 2113, Australia

^3^Brazilian Centre for Physics Research (CBPF), Rua Dr. Xavier Sigaud - 150, Urca, Rio de Janeiro RJ, CEP 22290180, Brazil

^4^School of Chemistry, Physics and Mechanical Engineering, Queensland University of Technology, Brisbane QLD 4000, Australia

^5^CSIRO-QUT Joint Sustainable Processes and Devices Laboratory, Commonwealth Scientific and Industrial Research Organization, P.O. Box 218, Lindfield NSW 2070, Australia

*Corresponding author. E-mail: Kostya.Ostrikov@qut.edu.au (K. Ostrikov)

**Supplementary Figures**


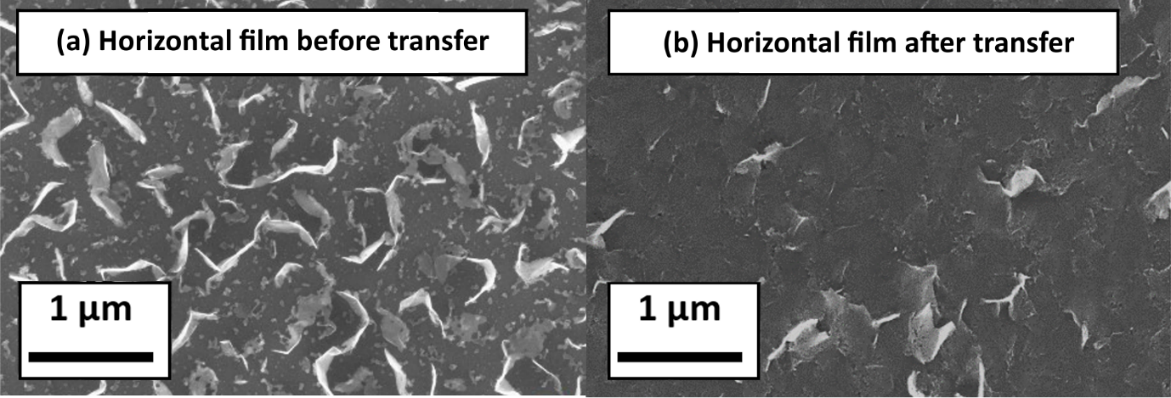


**Fig. S1** SEM images of the HGL sample prior the transfer from copper, produced with high hydrogen content, shows similar nanostructures to CNW. Image **a** shows visible edges perpendicular to the copper foil that collapse during the transfer process and form the horizontal graphene film **b**.


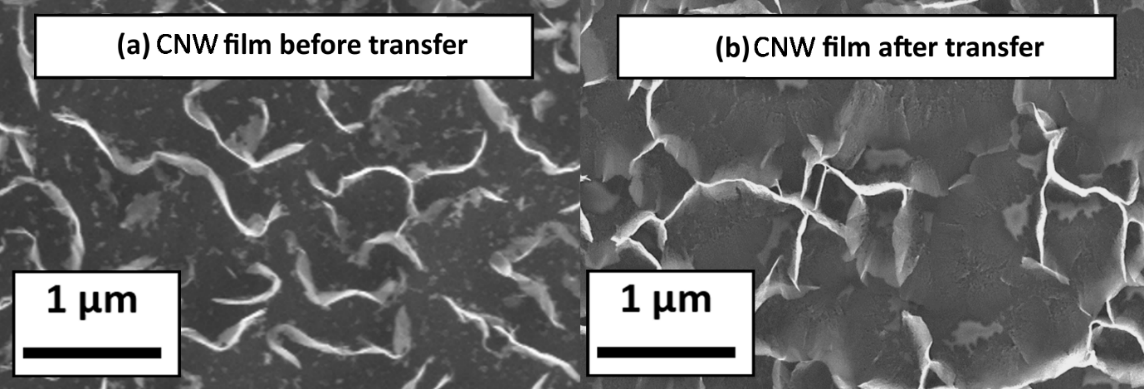


**Fig. S2** SEM images of the CNW sample prior the transfer from copper, produced with low hydrogen content. Image **a** shows dense edges perpendicular to the copper foil that stays during the transfer process to the coverslips **b**.


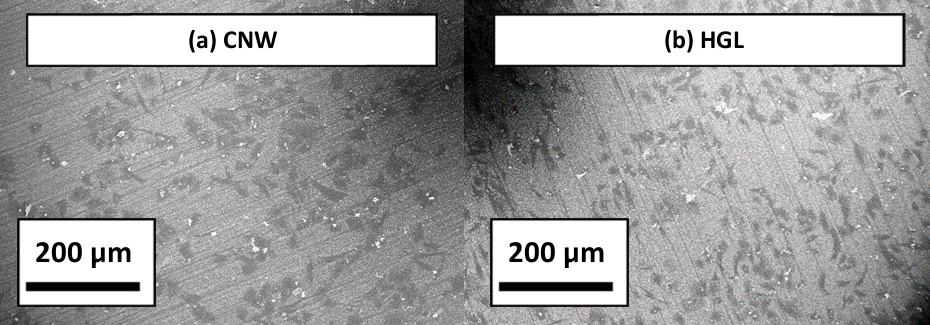


**Fig. S3** SEM images at a low magnification of **a** the CNW and **b** HGL samples showing similar amounts of proliferating cells.
